# Jejunal atresia type 4

**DOI:** 10.11604/pamj.2023.45.34.39092

**Published:** 2023-05-15

**Authors:** Lavanya Ramakrishnan, Revat Jagdish Meshram

**Affiliations:** 1 Datta Meghe Institute of Higher Education and Research, Wardha, India

**Keywords:** Neonate, congenital anomalies, intestinal atresias, acute abdomen, gastrointestinal tract malformations

## Image in medicine

Congenital gastrointestinal (GI) malformations like anorectal malformations (ARM), Hirschsprung's disease (HD), and intestinal atresias are conditions that are commonly presented. Though there is the possibility of some of them being detected antenatally in scans, they are usually missed or may even go undetected. Atresia is a congenital defect of a hollow viscus that results in complete obstruction of the lumen. Intestinal atresia is one of the most frequent causes of bowel obstruction in newborns and can occur at any point in the gastrointestinal tract. Cases of intestinal obstruction in neonates can be either congenital or acquired. Congenital causes involving obstruction of the digestive tract can be divided into high obstruction occurring above the level of the ileum and low-lying obstructions occurring in the ileum and the colon. Approximately one-half of the cases presenting with GI obstruction involve the duodenum. Amongst other congenital malformations of the GI tract, in the order of incidence, intestinal atresias are the most commonly presenting followed by anal atresia or anorectal malformations and lastly esophageal atresia as a part of the tracheo-esophageal fistula (TEF). Intestinal atresias contribute to nearly 50% of intestinal obstruction cases presenting in the neonatal period. About 20 percent of these are associated with chromosomal anomalies. Among the acquired causes of intestinal obstruction presenting in the neonatal period, midgut malrotation or midgut volvulus are seen, mostly in the first week of life. Here, we present a neonate brought to us on day 2 of life in view of feed intolerance at birth with bilious vomiting and abdominal distension. X-ray erect abdomen showed a classical triple bubble sign suggestive of jejunal atresia (A). The patient was taken up for explorative laparotomy and was found to have type high jejunal atresia with multiple, distal atretic segments, which falls into the type 4 category, the rarest form. Resection of 12 cm of the atretic jejunum and end-to-end anastomosis were done (B, C). All cases of obstruction, whether congenital or acquired, require radiological diagnosis as soon as one is suspected and surgical management in the neonatal period to avoid deleterious complications of gut ischaemia, perforation, and peritonitis. Most cases of neonatal GI obstruction have a good prognosis post-operatively, especially after the advent of parenteral nutrition with cautious its use and improved neonatal care facilities.

**Figure 1:**
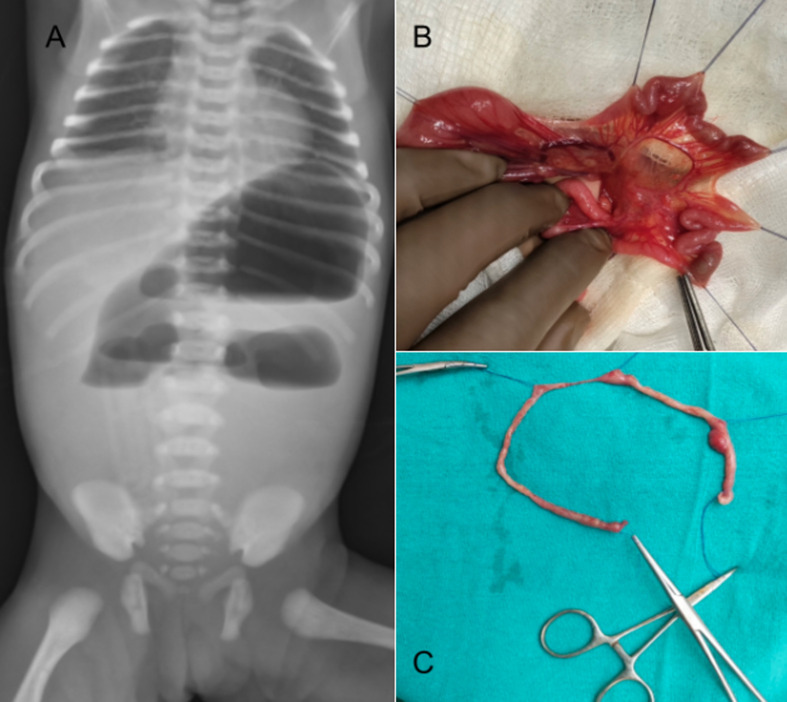
A) chest X-ray of the neonate showing a classical triple bubble sign; B,C) intraoperative photos showing multiple atretic segments of the jejunum

